# Simultaneous targeted and discovery-driven clinical proteotyping using hybrid-PRM/DIA

**DOI:** 10.1186/s12014-024-09478-5

**Published:** 2024-04-02

**Authors:** Sandra Goetze, Audrey van Drogen, Jonas B. Albinus, Kyle L. Fort, Tejas Gandhi, Damiano Robbiani, Véronique Laforte, Lukas Reiter, Mitchell P. Levesque, Yue Xuan, Bernd Wollscheid

**Affiliations:** 1 Institute of Translational Medicine (ITM), Department of Health Sciences and Technology (D-HEST), ETH Zurich, Zurich, Switzerland; 2 Swiss Institute of Bioinformatics (SIB), Lausanne, Switzerland; 3 ETH PHRT Swiss Multi-Omics Center (SMOC), Zurich, Switzerland; 4 Thermo Fisher Scientific (Bremen) GmbH, Bremen, Germany; 5 Biognosys, Schlieren, Switzerland; 6 Department of Dermatology, University Hospital Zurich, University of Zurich, Zurich, Switzerland

**Keywords:** Diagnostics, Clinical phenotyping, Melanoma, Proteotyping, Hybrid-PRM/DIA, Hybrid-DIA, Data-independent acquisition (DIA), Parallel reaction monitoring (PRM), Translational proteomics, Molecular tumor board

## Abstract

**Background:**

Clinical samples are irreplaceable, and their transformation into searchable and reusable digital biobanks is critical for conducting statistically empowered retrospective and integrative research studies. Currently, mainly data-independent acquisition strategies are employed to digitize clinical sample cohorts comprehensively. However, the sensitivity of DIA is limited, which is why selected marker candidates are often additionally measured targeted by parallel reaction monitoring.

**Methods:**

Here, we applied the recently co-developed hybrid-PRM/DIA technology as a new intelligent data acquisition strategy that allows for the comprehensive digitization of rare clinical samples at the proteotype level. Hybrid-PRM/DIA enables enhanced measurement sensitivity for a specific set of analytes of current clinical interest by the intelligent triggering of multiplexed parallel reaction monitoring (MSxPRM) in combination with the discovery-driven digitization of the clinical biospecimen using DIA. Heavy-labeled reference peptides were utilized as triggers for MSxPRM and monitoring of endogenous peptides.

**Results:**

We first evaluated hybrid-PRM/DIA in a clinical context on a pool of 185 selected proteotypic peptides for tumor-associated antigens derived from 64 annotated human protein groups. We demonstrated improved reproducibility and sensitivity for the detection of endogenous peptides, even at lower concentrations near the detection limit. Up to 179 MSxPRM scans were shown not to affect the overall DIA performance. Next, we applied hybrid-PRM/DIA for the integrated digitization of biobanked melanoma samples using a set of 30 AQUA peptides against 28 biomarker candidates with relevance in molecular tumor board evaluations of melanoma patients. Within the DIA-detected approximately 6500 protein groups, the selected marker candidates such as UFO, CDK4, NF1, and PMEL could be monitored consistently and quantitatively using MSxPRM scans, providing additional confidence for supporting future clinical decision-making.

**Conclusions:**

Combining PRM and DIA measurements provides a new strategy for the sensitive and reproducible detection of protein markers from patients currently being discussed in molecular tumor boards in combination with the opportunity to discover new biomarker candidates.

**Supplementary Information:**

The online version contains supplementary material available at 10.1186/s12014-024-09478-5.

## Background

To meet the demands of accurate diagnostics, it is essential to develop reliable and effective protein biomarkers that offer actionable information for disease diagnosis, prognosis, prediction, monitoring, and treatment stratification [1, 2]. Mass spectrometry (MS)-based proteomics can accurately identify and quantify thousands of potential marker proteins from a small, complex biospecimen simultaneously. Discovery-driven untargeted proteomics permits the identification of new marker proteins, post-translational modifications, and unbiased profiling of protein expression and expression changes. Nonetheless, untargeted acquisition schemes that use data-dependent acquisition (DDA) suffer from variable quantitative performance because of stochastic peptide selection for fragmentation [3, 4]. This has led to the development of methods that focus on improved stability and quantitative reproducibility, such as data-independent acquisition (DIA) [5–7]. DIA-based strategies offer the advantage of unbiased quantification of both peptide precursor (MS1 spectra) and fragment ions (MS2 spectra). Unlike DDA, which selects precursor ions based on intensity, DIA methods obtain information for all detectable ions in a given m/z range. The DIA data matrix is amenable to retrospective target, splice isoform, or PTM analysis [8]. This is particularly advantageous when studying clinical cohorts within a specific disease context, enabling researchers to identify potential novel biomarker candidates and unravel specific pathways beyond the initial data analysis. However, the combination of fragmentation in DIA has a drawback: high-abundance ions can mask fragmentation from less abundant species within a specific m/z isolation window. Consequently, low-abundant peptides might not be adequately sampled or could be masked by highly abundant signals [5]. This limitation reduces the dynamic range of DIA, particularly when the biological sample is complex and has a wide range of protein abundance, which in turn impairs the accurate detection of low-abundance biomarkers. As a result, clinical markers may be missed, or their abundance misestimated , hindering their successful translation and clinical application. To overcome these obstacles and improve the detection and reliable quantification of low-abundance proteins, it is possible to use targeted proteomics strategies. These strategies, which adopt a hypothesis-centered method, concentrate on identifying and quantifying particular proteins or peptides of interest. Selected/Multiple reaction monitoring (SRM/MRM) and parallel reaction monitoring (PRM) are examples of targeted acquisition schemes that provide high sensitivity and accuracy and allow for absolute quantification of disease markers [9–12]. Targeted acquisition strategies improve the reliability and sensitivity of biomarker detection, thereby facilitating their translation into routine clinical practice [13]. However, although elution time scheduling approaches can be applied, standard targeted acquisition schemes are typically limited in the number of peptides that can be monitored with enhanced detection and quantitation performance [14]. Acquisition methods that use spike-in references for triggering take a more selective approach by initiating MS acquisition only upon detection of a specific heavy reference peptide that co-elutes with the target peptide of interest. This selective triggering ensures that the MS acquisition is limited to the actual elution window of the peptide of interest, maximizing multiplexing capabilities and minimizing wasted scan time. Internal standard triggered-parallel reaction monitoring (IS-PRM) as one of the first spike-in triggering approaches was designed to maximize the effective time devoted to measuring the analytes in a time-scheduled targeted experiment [15]. Since then, several methods have been developed to improve this approach further, mainly by refining the acquisition algorithms implemented at the level of the mass spectrometer. Methods such as SureQuant [16, 17] or TOMAHAQ [18] employ an additional MS2-level check of the trigger spectrum as a selectivity filter to ensure that quantification scans are only triggered for authentic trigger peptide signals. Recently, hybrid-PRM/DIA was presented as an intelligent MS data acquisition strategy that combines DIA global proteome profiling with spike-in triggered multiplexed PRM (MSxPRM) for increasing the limit of detection and quantification of predefined targets in phosphoproteomics [19]. The authors benchmarked hybrid-PRM/DIA against SureQuant showing that both methodologies provide comparable quantification results concerning precision and accuracy [19]. Hybrid-PRM/DIA is particularly promising for the digitization of clinical sample cohorts as it provides clinically actionable information on marker proteins of interest through targeted acquisition and, at the same time, new research insights into disease development and treatment trajectories based on DIA. Clinical samples are valuable and often limited in quantity, making it important to get as much information as possible from a single measurement. Here we demonstrate the validity of the approach which we co-developed for the first time on patient samples and some clinically relevant markers in melanoma.

## Methods

### Sample preparation

All melanoma patient samples were collected according to the approval of the ethics commission (EK No. 647/800) and following the guidelines of the Helsinki Declaration on Human Rights. Melanoma patient samples from the Zurich URPP biobank [20] were processed using the Preomics iST kit (PreOmics). In short, samples were lysed at 95 °C for 10 min before sonication using three 30 s sonication pulses in a VialTweeter (Dr. Hielscher). Samples were digested for 3 h at 37 °C and peptides were further purified according to the manufacturer’s protocol. Thirty of these melanoma patient samples were monitored in hybrid-PRM/DIA (Additional file 2: Table S1).

Pierce HeLa Protein Digest Standard (Thermo Fisher Scientific) was resuspended at 1 µg/µl concentration in HPLC-grade water containing 0.1% (v/v) formic acid. Heavy as well as light SpikeTides™ sets for the detection of the most relevant tumor-associated antigens (TAAs) [21]) were derived via JPT technologies. For 65 of these TAAs, JPT synthesized proteotypic peptides with well-documented MRM transition data available (www.srmatlas.org, ISB), resulting in a set of 252 proteotypic peptides for monitoring (Additional file 2: Table S2). Peptides were resuspended at an approximate concentration of 50–150 pmol in a volume of 100 μl 10% ACN, 0.1% formic acid (approximate concentration of 1 pmol/μl per peptide). For dilution series measurements, the heavy reference peptides were kept constant in all samples at approximately 100 femtomoles (injected onto column). Their light counterparts were measured in a dilution series ranging from approximately 100 femtomoles to a minimum of 10 attomoles at most. Pierce HeLa Protein Digest Standard was used at a concentration of 0.5 μg/ μl as a background matrix in most of the measurements.

30 heavy reference AQUA peptides (Thermo Fisher Scientific, Synpeptide) for 28 proteins or 27 protein groups (Additional file 2: Table S3) were resuspended at a stock concentration of 10 pmol/μl. For triggering MSxPRM in hybrid-PRM/DIA, AQUA peptides were spiked into the melanoma patient samples at 50 fmol/μl. 2 μl of each sample were loaded onto the LC column for analyses. All samples were supplemented with synthetic retention time peptides (Biognosys) at a ratio of 1:20 v/v.

### Data acquisition

Samples were separated with a Thermo Scientific Easy-nLC 1000 using a 50 cm C18 EASY-Spray™ HPLC column (particle size 2 µm, 100 Å, 75 µm inner diameter, ES903, Thermo Fisher Scientific) (datasets Fig. 3, Additional file 1: Fig. S1) or a Vanquish™ Neo UHPLC system equipped with a 50 cm μPAC™ Neo HPLC column (bed length 50 cm, bed width 180 μm, COL-nano050NeoB, Thermo Fisher Scientific) (dataset Figs. 2, 4). For LC–MS/MS analyses performed on the Easy-nLC 1000 equipped with an ES903A, samples were loaded at 800 bar with 100% mobile phase A (99.9%H_2_O, 0.1%FA). Peptide elution was performed using a stepped gradient from 3 to 25% mobile phase B (99.9%ACN, 0.1%FA) in 90 min and 25% to 50% mobile phase B in 30 min. The flow rate was set to 200 nl/min. For LC–MS/MS analyses performed on the Vanquish™ Neo UHPLC system with a 50 cm μPAC™ Neo HPLC column, samples were loaded at 0.7 µl/min with 100% mobile phase A (99.9%H_2_O, 0.1%FA) with a pressure limit of 400 bar. The loading volume was set to automatic. Peptides were eluted with a stepped gradient of 5% to 32% mobile phase B (80%ACN, 0.1%FA) in 50 min and 32% to 60% mobile phase B in 10 min. The flow rate was set to 300 nl/min.

Samples were measured as replicates on an Orbitrap Exploris™ 480 mass spectrometer with an API providing hybrid-PRM/DIA capabilities (Thermo Fisher Scientific). DIA MS1 full scans were acquired with a resolution of 60,000 m/z. The normalized AGC target was set to 1000%, with maximum injection time to auto. DIA MS2 scans were performed over a scan range of 400–1210 with a resolution of 30,000 m/z. One MS1 scan was recorded every 18 MS/MS scans and a total of 54 MS2 scans were acquired to cover the full scan range (three MS1 scans per cycle) [2]. MS2 AGC target value was set to 1000% with a maximum injection time of 54 ms, the isolation window was set to 15. PRM scans were recorded with identical MS1 parameters as DIA. The number of multiplexed ions was set to two (heavy/light together), and the isolation window was 1.4 m/z. The resolution was 30,000 m/z, normalized AGC target was 1000%, and the maximum injection time was 54 ms. The scheduled isolation list for the 185 SpikeTides™ TAA heavy and light peptides was provided as a mass list table (Additional file 2: Table S4). Hybrid-PRM/DIA scans were triggered via an application programming interface (API) tool (moonshot_v1.3 or higher) (Thermo Fisher Scientific). The theoretical mass-to-charge value, charge state, and retention time window of internal standard peptides (parent ions) and corresponding endogenous peptides, as well as the theoretical mass-to-charge values of the fragments of internal standard peptides, were provided as an input.txt files (Additional file 2: Tables S5-S9). The following parameters were selected: 116 to 200 ms of maximum injection time, 10 ppm of mass error, MS intensity threshold of 1e5 or 5e4, and an AGC target of 1e6. Dynamic exclusion was set to 5–20 s.

PRM calibration curve measurements of the melanoma AQUA peptides were performed on an Orbitrap Exploris™ 480 mass spectrometer (Thermo Fisher Scientific) equipped with a 50 cm μPAC™ Neo HPLC column (COL-nano050NeoB, Thermo Fisher Scientific). Peptides were eluted with the same gradient as above. MS1 full scans were acquired with a resolution of 60,000 m/z over a scan range of 380–985 m/z. tMSn scans were acquired at R = 30,000 with an AGC target value set to standard and with a maximum injection time of 116 ms. HCD collision energy was set to 30%, and scheduling windows for the PRM scans were 6 min (Additional file 2: Table S10).

### Data analysis

For DIA analysis, files were searched in directDIA mode using Spectronaut v17 or higher (Biognosys). DIA scans from hybrid-PRM/DIA were extracted using the HTRMS converter tool from Spectronaut (v15.4 or higher), indicating Hybrid DIA conversion in conversion type. MS1 values were used for the quantification process, peptide quantity was set to mean. Data were filtered using Qvalue sparse with a precursor and a protein Qvalue cut-off of 0.01 FDR. Interference correction and local cross-run normalization were performed. MSxPRM data analysis from hybrid-PRM/DIA acquisitions was performed in SpectroDive™ (v11.3 and higher) (Biognosys) and partially Skyline (v20.2). For data analysis in SpectroDive, hybrid-PRM/DIA files were mostly converted into bgms format using the HTRMS converter in Hybrid DIA conversion mode. Experiment-specific panels were assigned. A spike in workflow was chosen for panel generation and Arg10 and Lys8 were defined as labelling channels. BGS Factory settings were set to default with a Qvalue cut-off of 0.01 FDR. Standard PRM data on the TAA jpt peptides as well as on the melanoma AQUA peptides were also analyzed and visualized using Skyline (v20.2).

## Results

### Targeted MSxPRM proteome profiling performance in hybrid-PRM/DIA

**Fig. 1:**
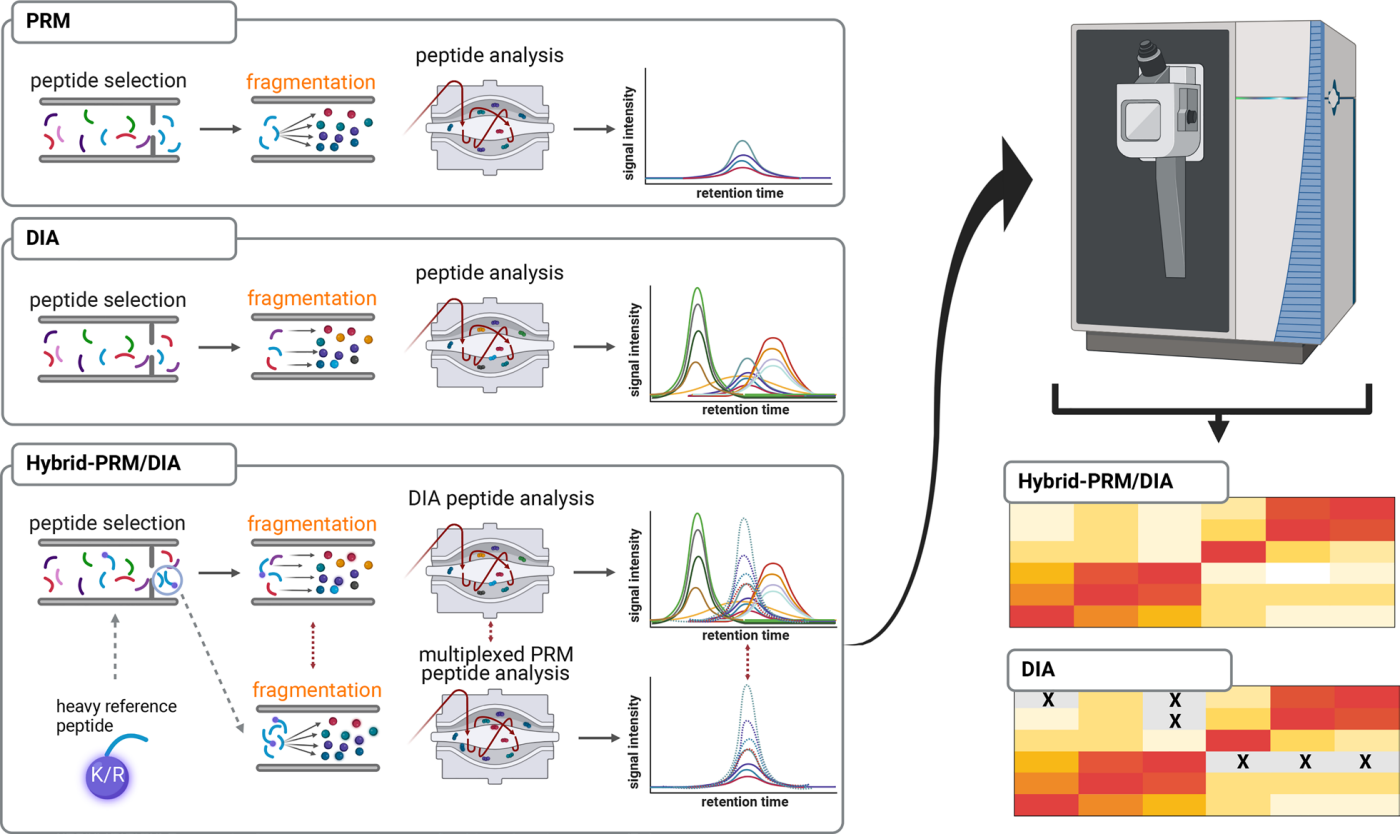
The hybrid-PRM/DIA acquisition scheme. Compared to conventional Parallel Reaction Monitoring (PRM) and Data-Independent Acquisition (DIA) methods, the hybrid-PRM/DIA approach utilizes rapid and simultaneous multiplexing of PRM MS/MS scans (MSxPRM) triggered by the detection of isotope-labeled heavy reference peptides. Successful detection of the isotope-labeled peptide triggers the high-quality measurement of the corresponding endogenous counter-peptide multiplexed with the isotope-labeled peptide by MSxPRM MS/MS scans. These scans are acquired with a narrower isolation window and maximized ion injection time for each species resulting in a higher sensitivity. Hybrid-PRM/DIA provides a data matrix with no missing data points of clinically relevant markers (heatmap gray: missing value; heatmap white: below LOD)

Hybrid-PRM/DIA was co-developed with Thermo Fisher Scientific to enhance the sensitivity of detection of selected predetermined peptides, while simultaneously producing exploratory-type data. The capability to obtain both types of data within a single measurement is especially crucial for clinical samples, where sample quantities are often limited. In the developed hybrid-PRM/DIA acquisition scheme, multiplexed MSxPRM MS/MS scans are triggered and integrated with Data Independent Acquisition (DIA) data (Fig. 1). The triggering of MSxPRM scans relies on identifying isotope-labeled heavy reference peptides. Successful identification of these labeled peptides enables the accurate quantification of their corresponding endogenous counterparts utilizing narrower isolation windows and optimized ion injection times for the different species. The hybrid-PRM/DIA acquisition strategy generates a comprehensive data matrix of DIA data points complemented by targeted peptide monitoring. This data matrix is particularly useful for proteotyping clinical biospecimens because it does not have any measurement gaps related to clinically relevant pre-selected markers. This is important for their evaluation in molecular tumor boards.

**Fig. 2:**
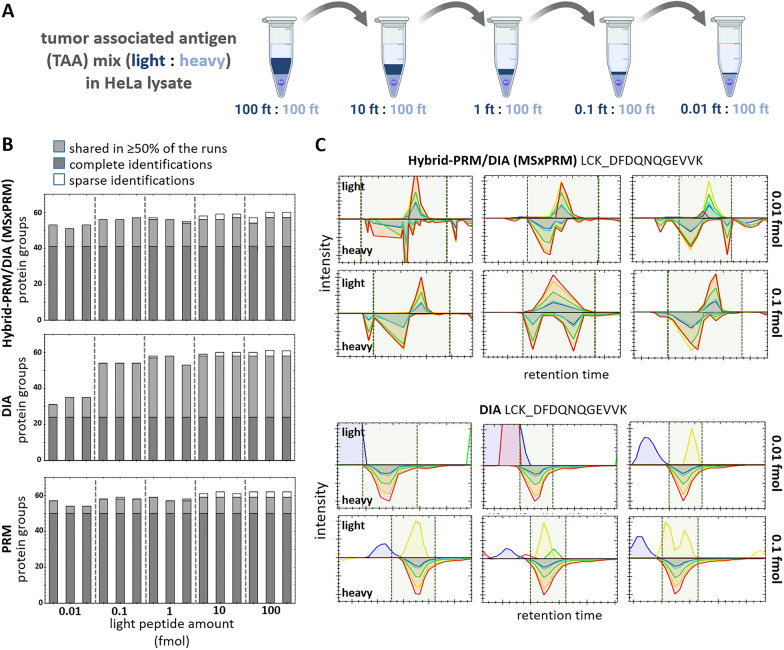
MSxPRM performance of hybrid-PRM/DIA on 185 TAA tumor-associated antigen peptides of 64 proteins. **A** Dilution series of the light TAA panel (approx. 0.01–100 fmol) in the heavy TAA reference (approx. 100 fmol, constant), which was used to trigger MSxPRM, and a HeLa digest as background matrix. **B** Number of identified protein groups in hybrid-PRM/DIA MSxPRM mode, DIA, and PRM for the different dilutions. Samples were measured as triplicates. **C** Hybrid-PRM/DIA MSxPRM and DIA measurements of the tyrosine-protein kinase Lck peptide DFDQNQGEVVK for 0.01 and 0.1 fmol. Shown are the intensities of the heavy and light transition peaks over three technical replicates

We initially evaluated the capacity of the hybrid-PRM/DIA technology using a set of 252 proteotypic peptides for a tumor-associated antigen (TAA) panel obtained from 65 annotated human proteins (Additional file 2: Table S1). These TAA peptide mixtures were available in both, their light and isotopically labeled heavy versions. We used the isotopically labeled peptides for triggering the MSxPRM scans in hybrid-PRM/DIA and the light peptides to simulate the endogenous peptide abundance of a potential sample. The heavy internal standard peptides were kept constant in all samples, maintaining a level of approximately 100 femtomoles (injected on the column). In contrast, the light counterparts underwent a dilution series ranging from roughly 100 femtomoles to 10 attomoles (Fig. 2A), reflecting the intensity range of endogenous peptides in different samples. After benchmarking the performance of our peptide mixes using MS/MS, we targeted a subset of high-quality 185 peptides associated with 64 proteins using MSxPRM, while simultaneously recording their DIA traces in hybrid-PRM/DIA. For DIA quantification we used our recently published MS1-based HRMS1-DIA approach [2]. On a high-resolution Orbitrap instrument, this approach results in an increased number of recorded MS1 scans to maximize peptide detection efficiency for more accurate quantification (three MS1 scans per cycle).

When comparing the identifications of light TAA peptides in hybrid-PRM/DIA MSxPRM mode to those of DIA and MSxPRM alone (multiplexed measurements of light and isotopically labeled counter-peptides) we observed that MSxPRM in hybrid-PRM/DIA generally yields superior signal-to-noise ratio and a lower limit of detection as compared to DIA alone (Fig. 2B, C with HeLa lysate background matrix, Additional file 1: Figs. S1A, S1B without HeLa lysate background matrix). This is particularly true within the lower abundance mass range of approximately 0.1 fmol and below. In the abundance range of about 10 attomoles, hybrid-PRM/DIA technology surpasses standard DIA by identifying an average of 52 protein groups versus 34 in DIA (Fig. 2B). Furthermore, the intelligent acquisition offers almost the same protein identification coverage and data completeness as the scheduled stand-alone PRM with an average of 55 protein groups (Fig. 2B). The performance of hybrid-PRM/DIA is further illustrated in Fig. 2C and Additional file 1: Fig. S1B, which show the detectability of the tyrosine-protein kinase Lck (LCK) peptide DFDQNQGEVVK for 0.01 and 0.1 fmol (Fig. 2B) and that of the melanoma-associated antigen 3 (MAGEA3) peptide ISGGPHISYPPLHEWVLR for 0.1 and 1 fmol (Additional file 1: Fig. S1B), comparing hybrid-PRM/DIA with DIA. The narrow isolation window and maximized ion injection time of the light peptides in the MSxPRM scan of hybrid-PRM/DIA helped improve the selectivity and sensitivity of quantification, as well as the detection reliability, specifically when the background noise was high.

**Fig. 3:**
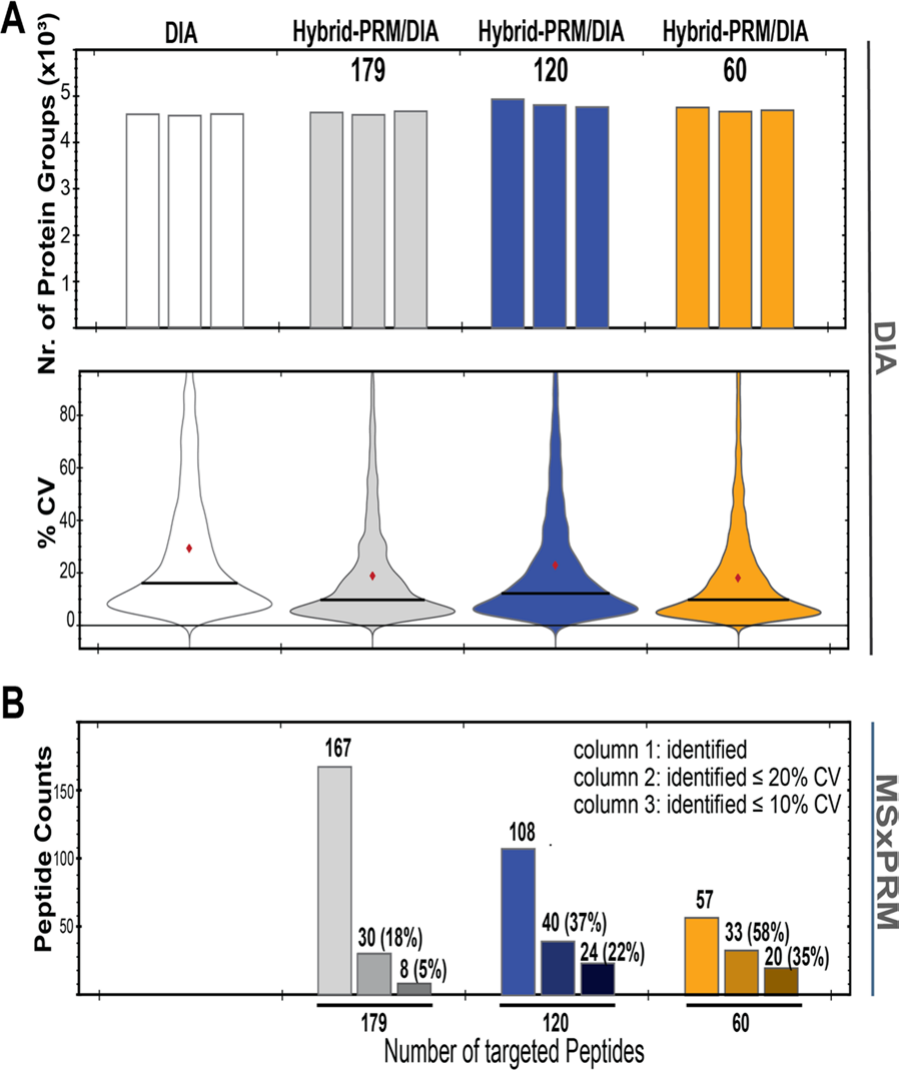
Benchmarking of DIA performance in hybrid-PRM/DIA with an increasing number of triggered MSxPRM scans. **A** Protein groups identified through DIA in hybrid-PRM/DIA with a predefined list of  TAA target peptides (179, 120, and 60 peptides) compared to standard DIA. The HeLa digest was spiked with a mixture of crude TAA heavy triggering peptides and TAA light peptides, both at an amount of  100 fmol per injection. The lower panel of Fig. A shows the median CV values for all proteins quantified in DIA and hybrid-PRM/DIA. **B** Identified peptides, identified peptides with CVs below 20% or 10% respectively are shown for the different numbers of hybrid-PRM/DIA peptide targets in MSxPRM mode

We also evaluated the hybrid-PRM/DIA globally for reproducibility. We determined the inter-injection median CVs over the three technical replicates measured at different concentrations. We found that the MSxPRM of the hybrid-PRM/DIA outperformed DIA in terms of CVs smaller than 20% only in the low abundance range (Additional file 1: Figs. S1C-F, S2A). However, the reproducibility of the measurements in scheduled stand-alone PRM and also in DIA on more abundant targets was better in terms of inter-injection median CVs. This is partly due to the thresholding of the MSxPRM event trigger, which sometimes led to a delay in signal acquisition and, thus, truncation of peaks for quantification. In addition, the number of MSxPRM scan events triggered in parallel also contributed to the rather high CV values we observed globally, caused by a considerable amount of time spent on the fast MSxPRM validation/triggering as well as the actual quantification MSxPRM scans (see also Fig. 3).

### Global DIA proteome profiling performance of hybrid-PRM/DIA

The global profiling performance of DIA in hybrid-PRM/DIA MS was investigated and compared with the standard stand-alone, high-resolution MS1-based DIA-MS method described above [2]. The analysis was conducted on a HeLa cell lysate digest spiked with the mixture of crude TAA panel light and heavy peptides at an approximate concentration of 100 fmol each. We evaluated the total protein group identifications for 0.5 μg of injected HeLa lysate by DIA (hybrid-PRM/DIA and stand-alone DIA) while simultaneously triggering scheduled MSxPRM scans of 60, 120, and 179 target peptides of the TAA panel in hybrid-PRM/DIA (Additional file 2: Tables S6-S8). Approximately 4600-4800 protein groups were identified at 1% FDR in a 120-min gradient on an ES903 50 cm C18 column in all DIA and hybrid-PRM/DIA conditions, regardless of the number of triggering events (Fig. 3A). This is in the same range as for the TAA dilution experiment on the μPAC™ Neo HPLC column (Additional file 1: Fig. S2B). Even when the number of target peptides was increased to 179, i.e. up to 36 parallel MSxPRM events (Additional file 1: Fig. S3), DIA data acquisition in hybrid-PRM/DIA demonstrated consistent and competitive proteome profiling capabilities. In addition to the similar number of identified protein groups, DIA in hybrid-PRM/DIA showed good quantification precision of proteins and protein groups with median CVs between 10% and 16% (Fig. 3A, bottom panel). In terms of target peptide identification, MSxPRM in hybrid-PRM/DIA was able to identify 57, 108, and 167 peptides, respectively, with a 1% FDR in SpectroDive (Fig. 3B). It can be seen that the coefficient of variation for peptide quantification increased with the number of targeted peptides: while 58% of the 60 peptides monitored had CVs ≤ 20%, this number decreased to only 18% when 179 peptides were monitored. This can be explained by the increasing number of parallel scheduling events and the increased time needed for the fast MSxPRM validation/triggering, as well as the actual quantification MSxPRM scans.

### Evaluation of hybrid-PRM/DIA on clinical samples using a melanoma diagnostic marker panel

Hybrid-PRM/DIA was applied to a set of de-identified tumor specimens from melanoma patients to obtain molecularly actionable data from limited biological samples while digitizing the proteotype for future research studies. Initially, the global proteotype of the cohort of 95 samples was determined by standalone DIA. Subsequently, the Hybrid-PRM/DIA technique was used on a subset of 30 of these samples to monitor clinically relevant level-1 and level-2 marker proteins more reliably. Level-1 proteins serve as diagnostic markers with immediate clinical relevance [22]. Level-2 protein information provides additional details on potential drug targets or pathway nodes. Furthermore, DIA-MS complemented the data matrix with level-3 information on the global proteotype. This enabled a comprehensive characterization of the clinical phenotype and may lead to the discovery of novel biomarkers in the future.

Our comprehensive level-1 and level-2 melanoma marker list consisted of 65 protein groups relevant to melanoma disease diagnosis and treatment decision-making (Additional file 2: Table S10) [23]. Of these 65 protein groups, 43 were detectable in our melanoma patient cohort using standard DIA (Additional file 1: Fig. S4, Additional file 2: Table S11) [2]. The extracted level-1 and level-2 marker proteins exhibited varying degrees of missing data, with an average of 34% missingness across the entire patient cohort and extracted protein data matrix. The highest degrees of missingness were observed for the cellular tumor antigen p53 (TP53) and the G1/S-specific cyclin-D3 (CCND3) (Additional file 1: Fig. S4). We then synthesized 30 AQUA peptides to specifically monitor 28 proteins from our panel in MSxPRM using hybrid-PRM/DIA (Additional file 2: Table S3). The list of AQUA peptides included peptides for the melanocyte protein PMEL as a diagnostic marker for melanocytic tumors [22] and the drug target cyclin-dependent kinases 4 CDK4 [24]. Melanoma is an attractive target for CDK4/6 inhibitors because the p16INK4a/Cyclin D1-CDK4/6/RB pathway is dysregulated in the majority of melanomas [25]. Also, the neurofibromin protein NF1 as a tumor suppressor was monitored. Targeting NF1-regulated pathways such as RAS/MAPK or PI3K/mTOR offers potential therapeutic options for patients with melanoma [26, 27]. Our panel also included a peptide of the tyrosine-protein kinase receptor UFO, which is encoded by the AXL gene. AXL expression has been suggested to be associated with epithelial-mesenchymal transition (EMT) in melanoma, which contributes to both metastatic spreading and therapy resistance in cancer [28]. In addition, receptor tyrosine kinase inhibition has recently been shown to improve BRAF-targeted therapy [29–31]. The degree of missingness for UFO in our standard DIA protein data matrix is quite high (80%), so targeting UFO is an ideal test case scenario to detect peptide amounts close to the limit of detection.

**Fig. 4:**
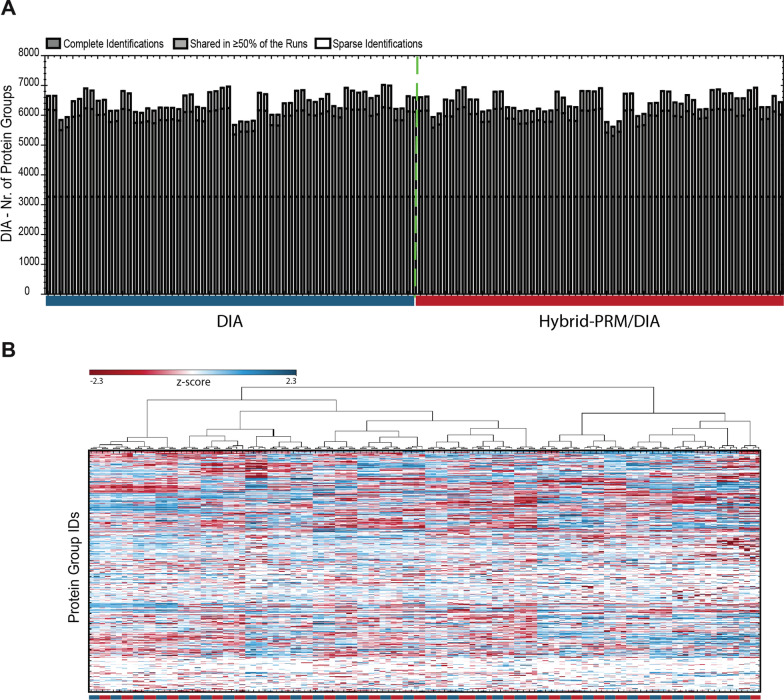
Identified protein groups by DIA or by DIA in hybrid-PRM/DIA in thirty melanoma patient samples. **A** The graph displays the number of identified protein groups per measurement for thirty melanoma patient samples. The samples were measured as technical replicates using DIA and hybrid-PRM/DIA. In **B**, the protein group measurements of DIA and hybrid-PRM/DIA are presented in a heatmap, clustered by hierarchical clustering (blue: DIA, red: hybrid-PRM/DIA)

**Fig. 5:**
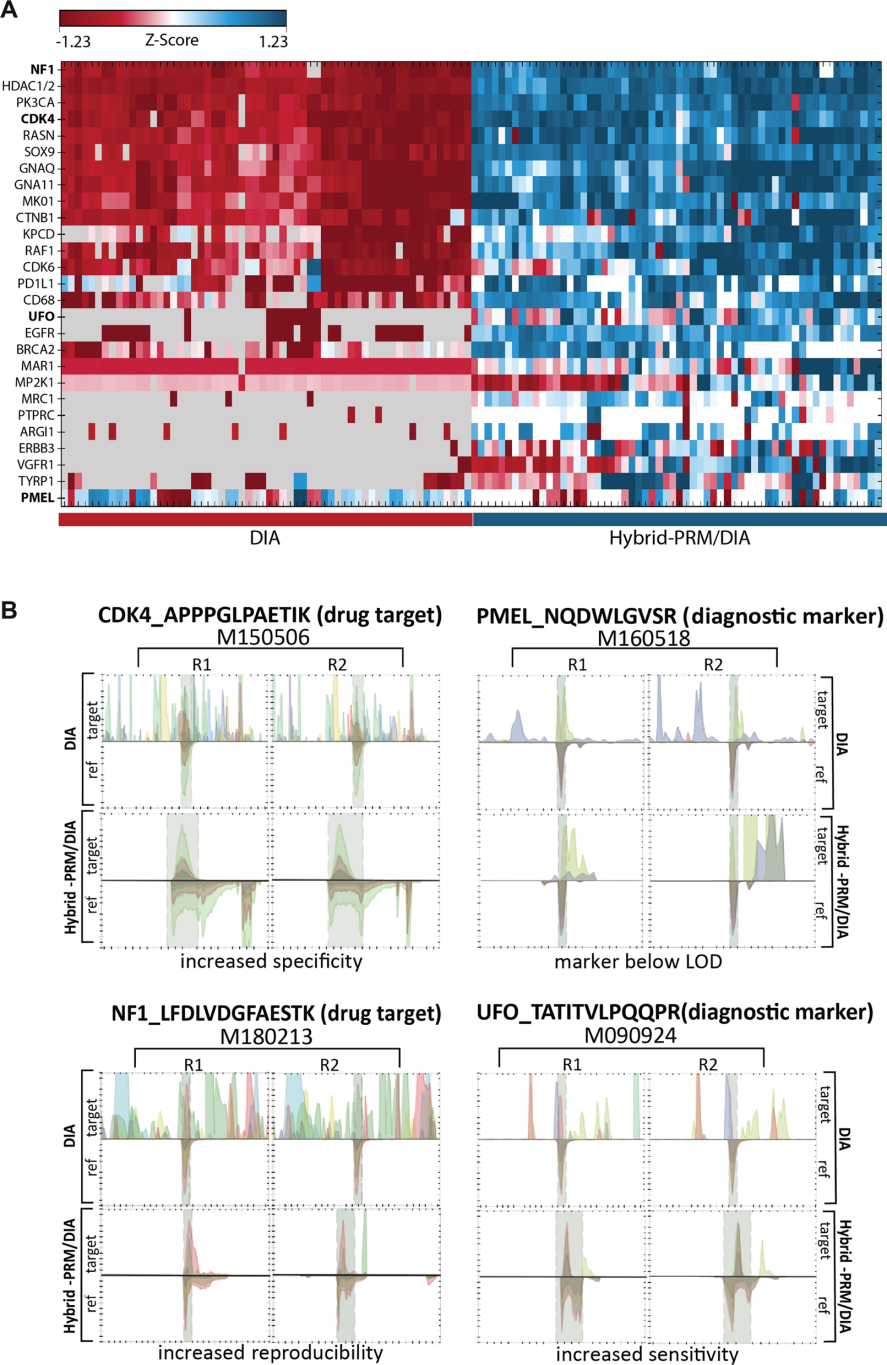
Monitoring of 27 melanoma-associated protein groups in thirty patient samples by DIA or Hybrid-PRM/DIA. **A** Heatmap of 27 protein groups that were detected and quantified by DIA or by MSxPRM of hybrid-PRM/DIA in 30 melanoma patient samples. Heavy reference peptides were used for triggering and peak integration. All samples were measured in duplicate for each acquisition scheme. Missing data points in DIA are shown in gray, values below the LOD in MSxPRM are shown in white. The four proteins CDK4, PMEL, NF1, and UFO shown in panel B are highlighted in bold. **B** Four examples illustrating the benefits of MSxPRM measurements in hybrid-PRM/DIA. MSxPRM helped to increase the specificity, reproducibility, and sensitivity of peptide detection. Peptide levels below the limit of detection (LOD) could be unambiguously assigned (target: light endogenous peptide, ref: heavy reference peptide)

We benchmarked the performance of hybrid-RPM/DIA versus DIA by measuring technical replicates on the subset of 30 patient samples where enough material was available. The 30 AQUA peptides were used as triggers for MSxPRM. DIA in hybrid-PRM/DIA led to an overall identification of close to 6500 protein groups, which is in the same range as for DIA alone (Fig. 4A). The measured samples clustered nicely by technical replicate and acquisition scheme (Fig. 4B). When we extracted the peptide traces for the 30 AQUA peptides, we found that the data completeness was 70.1% for the monitored peptides in DIA, compared to 84.4% in hybrid-PRM/DIA (Fig. 5A). In hybrid-PRM/DIA, the spike-in reference allowed us to clearly assign the 15.6% of missing values as below the limit of detection, excluding technical artifact as a reason for data missingness. The narrow isolation window and maximized ion injection time of the hybrid-PRM/DIA MSxPRM scans improved the selectivity and sensitivity of quantification, as shown for CDK4 in patient sample M150506 and for UFO in patient sample M090924 (Fig. 5B). The MS2 traces of the NF1 peptide LFDLVDGFAESTK in M180213 showed a more reproducible extraction in hybrid-PRM/DIA than in DIA (Fig. 5B). Specifically, we observed that in hybrid-PRM/DIA detection and quantification reliability increases in areas with a high background. Hybrid-PRM/DIA also allows for determining the lower limit of detection of endogenous peptides based on the spiked heavy-labeled reference. For instance, in the case of PMEL, we clearly showed that the endogenous peptide was not detectable in patient sample M040418 because it was below the detection limit (< 0.0101 fmol/μl) as estimated from the heavy reference calibration curve (Additional file 1: Fig. S5). This helps to avoid biases in downstream data processing, such as those introduced by imputation algorithms, which could lead to misinterpretation of the results. In conclusion, we have demonstrated that the new hybrid-PRM/DIA strategy has the potential to monitor clinical marker peptides with greater reliability, sensitivity and specificity than DIA alone, and after formal assay development and qualification, even allows absolute quantification of the monitored endogenous peptides. Additionally, the global proteotype can be monitored equally well in hybrid-RPM/DIA as in DIA.

## Discussion

Here, we present the newly developed hybrid-PRM/DIA technology as a suitable approach for reproducibly monitoring clinical protein markers while simultaneously generating data for discovery-driven research. The hybrid-PRM/DIA strategy provides scalability in terms of productivity and turnaround time. By comparing hybrid-PRM/DIA with standalone DIA, we found that hybrid-PRM/DIA can improve signal-to-noise ratios, increase the limit of detection and quantitation, reduce interferences, and achieve greater precision in quantitation for lower concentrations. Hybrid-PRM/DIA methods provide equivalent proteome profiling capabilities compared to standard DIA methods for DIA-driven proteomics discovery. Hybrid-PRM/DIA methods also demonstrate robust precision in protein and peptide quantification in DIA, with median CVs ranging from 10 to 16%. The data presented here shows that 60–179 peptides can be targeted concurrently in MSxPRM without compromising DIA acquisition.

We demonstrated the usefulness of hybrid-PRM/DIA on a melanoma patient cohort, expanding on the study of the Olsen lab showing the impact of the strategy on phospho-signaling differences [19]. Here, we could show that we can generate clinically informative data in a reliable and if required, even absolute quantitative fashion upon assay qualification. Sensitivity in hybrid-PRM/DIA is increased due to narrow isolation windows and increased ion injection times in MSxPRM, resulting in better signal-to-noise ratios. Weak signals of endogenous peptides can be identified based on the spike-in reference, even if only a few fragment ions are detectable. Retention time alignment and co-isolation with the peptide's heavy counterpart boost the signal in terms of annotation and identification. Altogether, this helps to avoid missing values in a quantitative protein data matrix. If the endogenous peptide is not identified along with its heavy counterpart, it is considered to be below the limit of detection. The limit of detection can be determined by recording dilution series and calibration curves.

One of the challenges of the hybrid-PRM/DIA method at this stage is peak clipping, mainly due to the lack of an API option to define a signal-to-noise ratio for triggering the MSxPRM scan. In some of the measured cases, we observed a delayed triggering of the MSxPRM event, resulting in only partial capture of the endogenous peptide signal. This is something that will be addressed in the updated release of the API, which will lead to a further reduction in CVs in MSxPRM.

## Conclusions

Hybrid-PRM/DIA enables the selected and comprehensive digitization of precious clinical samples that cannot be regenerated. The presented MS acquisition strategy further provides quantitative clinically relevant information on endogenous protein abundances. This PRM-based information can provide clinicians with protein information useful for discussions and clinical decision support in molecular tumor boards today. However, from a translational perspective, the DIA information generated in parallel from the same clinical biospecimen provides a wealth of research data for the development of theranostic strategies benefiting patients tomorrow.

### Supplementary Information

**Additional file 1: Fig. S1.** MSxPRM performance of hybrid-PRM/DIA on tumor-associated antigen peptides without HeLa background matrix.** Fig. S2.** Hybrid-PRM/DIA performance on TAA peptides in terms of CVs (MSxPRM) and protein group identifications (DIA).** Fig. S3. **Scheduling windows for monitoring 60, 120 or 179 targets simultaneously in MSxPRM.** Fig. S4. **Extracted level-1/level-2 melanoma markers from a DIA data matrix of 95 melanoma patient samples.** Fig. S5.** Calibration curve of the PMEL peptide NQDWLGVSR

**Additional file 2: Table S1.** List of biobanked melanoma patient samples monitored with hybrid-PRM/DIA and DIA.** Table S2.** Overview of jpt TAA peptide panel (heavy and light).** Table S3.** List of 30 AQUA peptides used as melanoma level-1/2 diagnostic markers.** Table S4.** PRM scheduling list of the 185 TAA peptides plus 11 retention time peptides.** Table S5.** Hybrid-PRM/DIA API input list for triggering of MSxPRM scans of the 185 TAA peptides.** Table S6.** Hybrid-PRM/DIA API input list for triggering of MSxPRM scans of the 179 TAA peptides.** Table S7.** Hybrid-PRM/DIA API input list for triggering of MSxPRM scans of the 120 TAA peptides.** Table S8.** Hybrid-PRM/DIA API input list for triggering of MSxPRM scans of the 60 TAA peptides.** Table S9.** Hybrid-PRM/DIA API input list for triggering of MSxPRM scans of the 30 melanoma AQUA peptides.** Table S10.** PRM scheduling list of the 30 melanoma AQUA peptides plus 11 iRT peptides.** Table S11.** List of the 65 level-1 and level-2 melanoma marker proteins/protein groups.** Table S12.** Quantitative protein matrix over 43 melanoma marker proteins in 95 patient samples. Values are log10-transformed and the basis of the heatmap in Fig. S4. The proteins discussed in Fig. 5B are indicated in red.

## Data Availability

The mass spectrometry data (raw files) were deposited in the MassIVE dataset repository with the dataset identifier MSV000092996. The application programming interface (API) tool is available at https://github.com/thermofisherlsms/MoonshotApps.

## Abbreviations

API: Application programming interface
AQUA: Absolute QUAntification
CDK4: Cyclin-dependent kinases 4
LOD: Limit of detection
MSxPRM: Multiplexed parallel reaction monitoring
NF1: Neurofibromin
PMEL: Melanocyte protein PMEL
TAA: Tumor associated antigen
TP53: Cellular tumor antigen p53
UFO/AXL: Tyrosine-protein kinase receptor UFO
