# Supplementary material for: Simultaneous targeted and discovery-driven clinical proteotyping using hybrid-PRM/DIA
Source: Clin Proteomics. 2024 Apr 2;21:26. doi: 10.1186/s12014-024-09478-5 (PMC10988896; doi:10.1186/s12014-024-09478-5)
Supplement: Supplementary file 1 — Additional file 1: Fig. S1. MSxPRM performance of hybrid-PRM/DIA on tumor-associated antigen peptides without HeLa background matrix. Fig. S2. Hybrid-PRM/DIA performance on TAA peptides in terms of CVs (MSxPRM) and protein group identifications (DIA). Fig. S3. Scheduling windows for monitoring 60, 120 or 179 targets simultaneously in MSxPRM. Fig. S4. Extracted level-1/level-2 melanoma markers from a DIA data matrix of 95 melanoma patient samples. Fig. S5. Calibration curve of the PMEL peptide NQDWLGVSR [file 12014_2024_9478_MOESM1_ESM.docx]

#### **Supporting Information**

#### **Supplementary Methods**

#### **DIA measurement and data analysis scheme for 95 sample cohort proteotpying (Fig. S4)**

Samples were analyzed on a Q Exactive HFX mass spectrometer (Thermo Fisher Scientific) equipped with an Easy-nLC 1200 (Thermo Fisher Scientific). Peptides were separated on an Acclaim PepMap RSLC C18, 2 µm, 100 Å, 150 µm i.d. x 150 mm, nanoViper EASY-Spray column (ES806, Thermo Fisher Scientific). Mobile phase A consisted of HPLC-grade water with 0.1% formic acid, and mobile phase B consisted of HPLC-grade ACN (80%) with HPLC-grade water and 0.1% (v/v) formic acid. Peptides were eluted at a flow rate of 1200 nl/min using a stepped gradient from 2% to 8% mobile phase B in 4 min, 8% to 32% in 49 min and 32% to 60 % in 1 min. MS1 in DIA covered a mass range of m/z 400-1210, MS1 was recorded at a resolution of 120,000 with an AGC target of 3e6 and a maximum injection time of 50 ms. The DIA isolation window size was set to 15 m/z and a total of 54 DIA scan windows were recorded at 30,000 resolution with an AGC target value of 1e6 and a loop count of 18 [[2]](https://paperpile.com/c/mOQgXk/Oneu). HCD fragmentation was set to 28 normalized collision and default charge state 3 with a starting m/z of 200.

DIA data were analyzed using Spectronaut v12 (Biognosys). MS1 values were used for the quantification, peptide quantity was set to sum. Data were filtered using Qvalue (identified) with a precursor and a protein Qvalue cut-off of 0.01 FDR. Interference correction as well as local cross-run normalization was performed.

**Supplemental Tables**

Datasheets table S1-S12 are merged in a combined excel file.

**Table S1.** List of biobanked melanoma patient samples monitored with hybrid-PRM/DIA and DIA.

**Table S2.** Overview of jpt TAA peptide panel (heavy and light).

**Table S3.** List of 30 AQUA peptides used as melanoma level-1/2 diagnostic markers.

**Table S4.** PRM scheduling list of the 185 TAA peptides plus 11 retention time peptides.

**Table S5.** Hybrid-PRM/DIA API input list for triggering of MSxPRM scans of the 185 TAA peptides.

**Table S6.** Hybrid-PRM/DIA API input list for triggering of MSxPRM scans of the 179 TAA peptides.

**Table S7.** Hybrid-PRM/DIA API input list for triggering of MSxPRM scans of the 120 TAA peptides.

**Table S8.** Hybrid-PRM/DIA API input list for triggering of MSxPRM scans of the 60 TAA peptides.

**Table S9.** Hybrid-PRM/DIA API input list for triggering of MSxPRM scans of the 30 melanoma AQUA peptides.

**Table S10.** PRM scheduling list of the 30 melanoma AQUA peptides plus 11 iRT peptides.

**Table S11.** List of the 65 level-1 and level-2 melanoma marker proteins/protein groups.

**Table S12.** Quantitative protein matrix over 43 melanoma marker proteins in 95 patient samples. Values are log10-transformed and the basis of the heatmap in Fig. S4. The proteins discussed in Fig. 5B are indicated in red.

#### **Supplemental Figures**


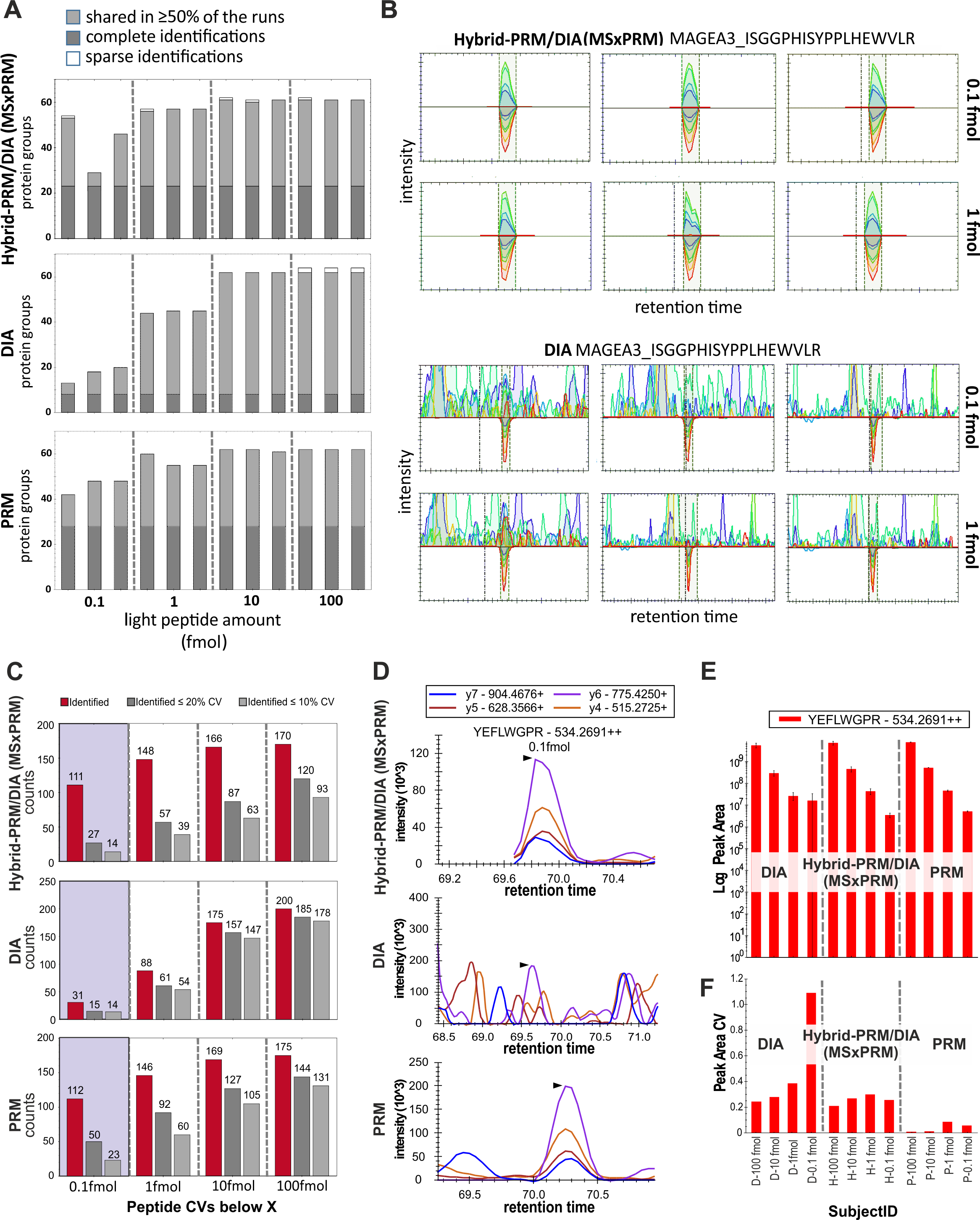


##### Fig. S1. MSxPRM performance of hybrid-PRM/DIA on tumor-associated antigen peptides without HeLa background matrix.

**(A)** Number of identified protein groups in hybrid-PRM/DIA MSxPRM mode, DIA, and PRM for four different dilutions (approx. 0.1-100 fmol). Samples were measured as triplicates. **(B)** Hybrid-PRM/DIA MSxPRM and DIA measurements of the Melanoma-associated antigen 3 (MAGEA3) peptide ISGGPHISYPPLHEWVLR for 0.1 fmol and 1 fmol. Shown are the heavy and the light transition traces over three technical replicates. **(C)** The number of identified peptides in red is plotted against the number of identified peptides at a CV ≤ 20% (dark gray) and at a CV ≤ 10% (light gray). Shown are averages over three technical replicates for the range of 0.1 fmol to 100 fmol. Transitions for one replicate at 0.1 fmol **(D)**, log peak areas **(E)**, and peak area CVs over three replicates **(F)** of the dilution series are depicted for one additional example, i.e. the peptide YEFLWGPR of the Melanoma-associated antigen C1 (MAGC1_HUMAN).


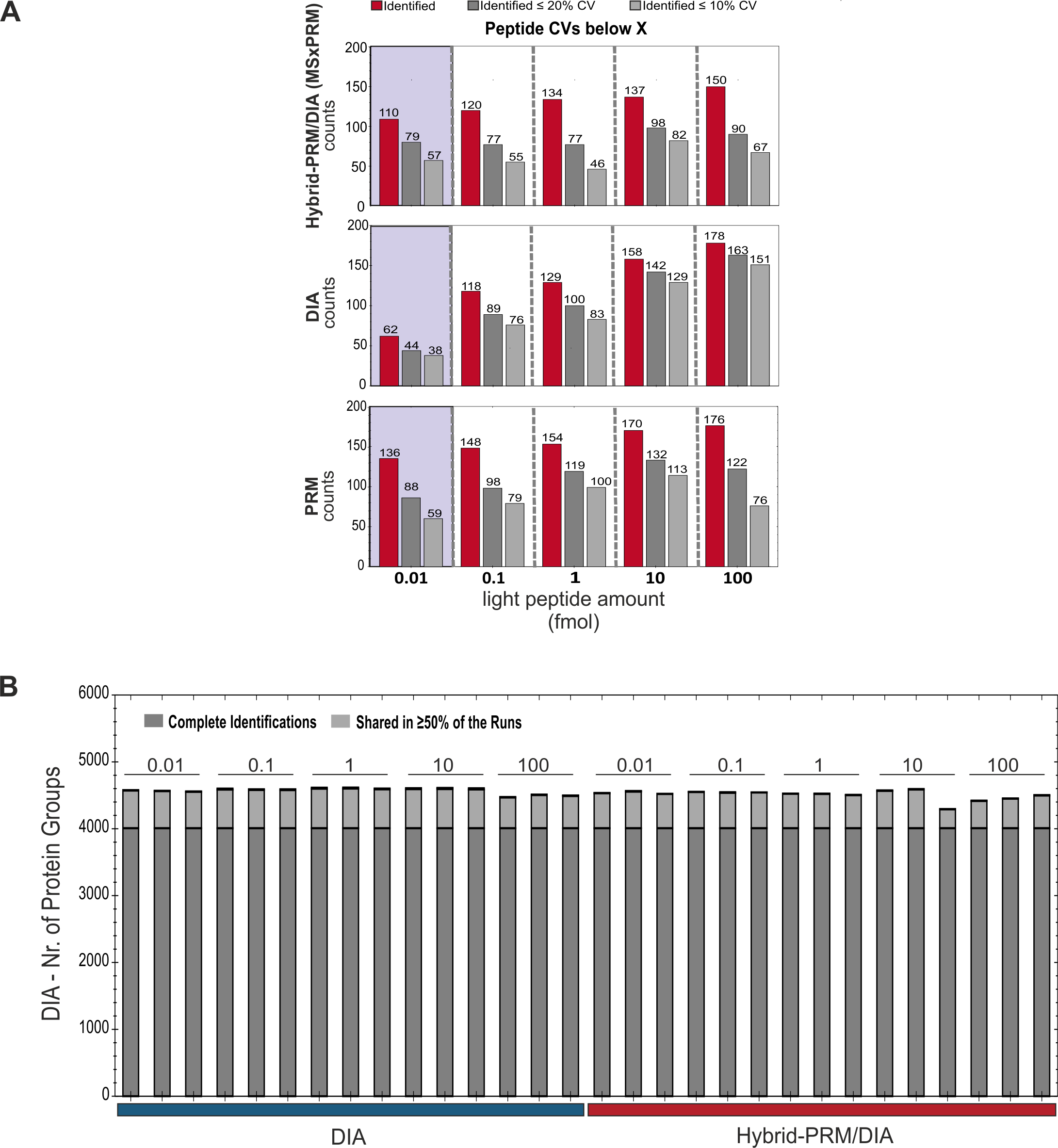


##### Fig. S2. Hybrid-PRM/DIA performance on TAA peptides in terms of CVs (MSxPRM) and protein group identifications (DIA).

**(A)** The number of identified peptides in red is plotted against the number of identified peptides at a CV ≤ 20% (dark gray) and at a CV ≤ 10% (light gray). Shown are averages over three technical replicates for the range of 0.01 fmol to 100 fmol. **(B)** Number of identified protein groups by DIA versus DIA in hybrid-PRM/DIA. All samples were measured in triplicate.

#####
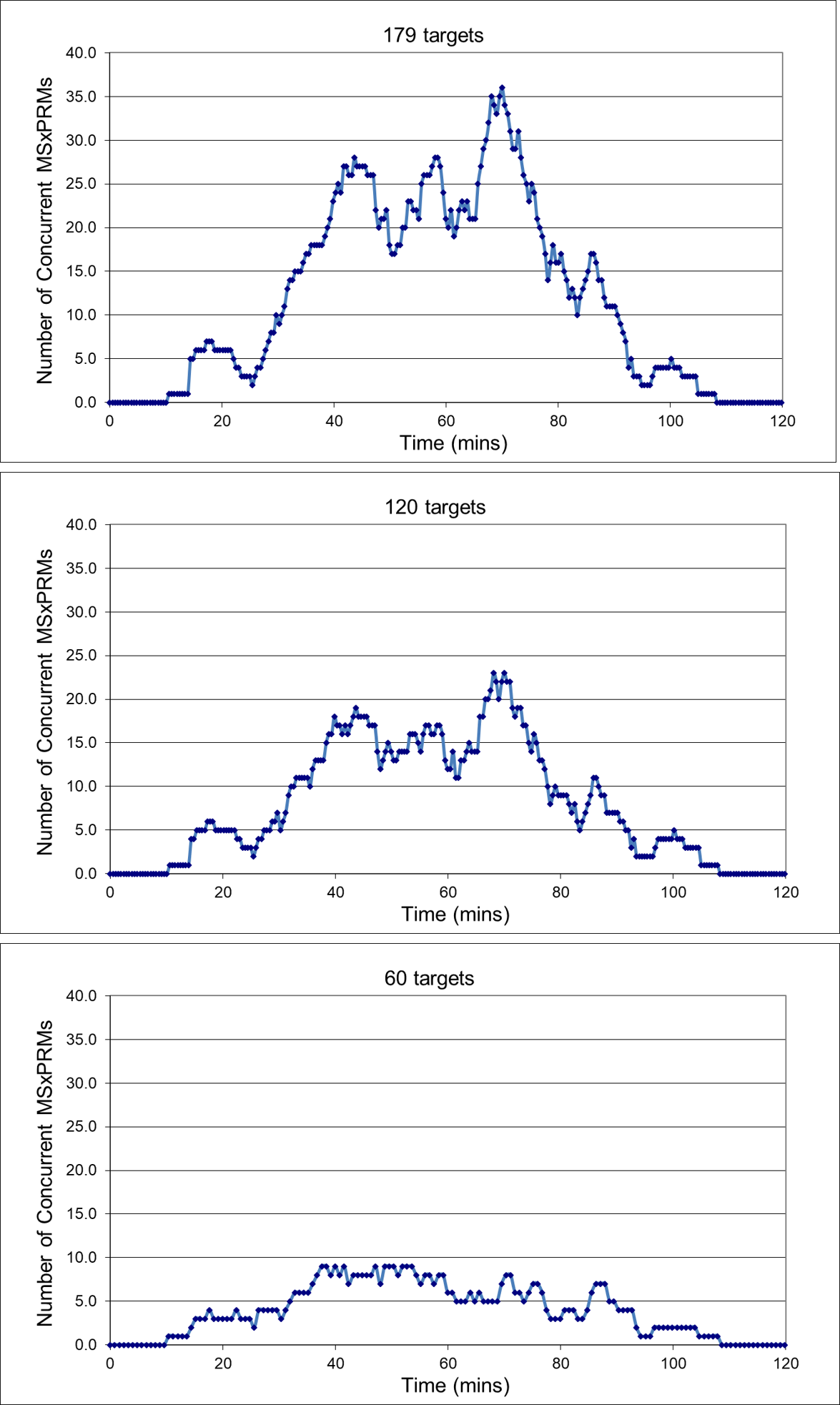


##### Fig. S3. Scheduling windows for monitoring 60, 120 or 179 targets simultaneously in MSxPRM.

Shown is the number of concurrent MSxPRM scheduling windows over retention time. For 179 targets, this number can be as high as 36 synchronously monitored peptides.


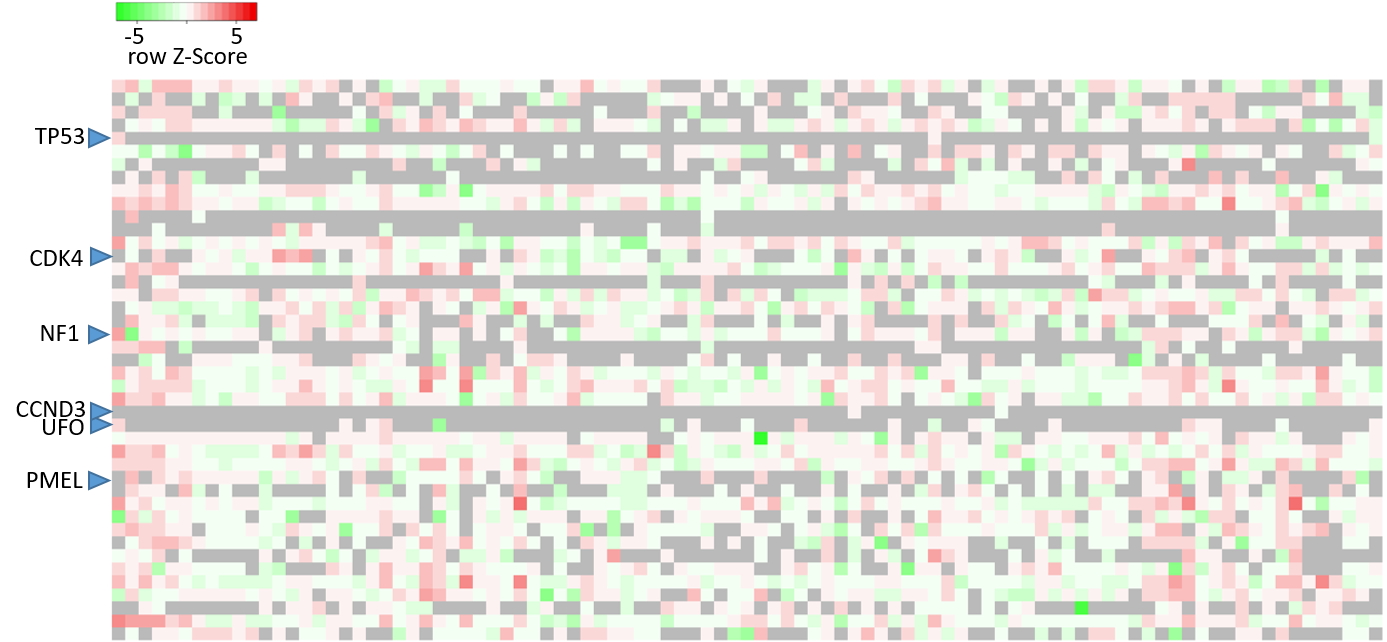


##### Fig. S4. Extracted level-1/level-2 melanoma markers from a DIA data matrix of 95 melanoma patient samples.

The heatmap shows 43 protein groups that were repetitively detected in a biobanked melanoma cohort of 95 patients in standard DIA. The four proteins CDK4, NF1, UFO, and PMEL shown in Fig. 4 as well as TP53 and CCND3 discussed in the manuscript are indicated with an arrow. Missing values in the data matrix are depicted in gray.


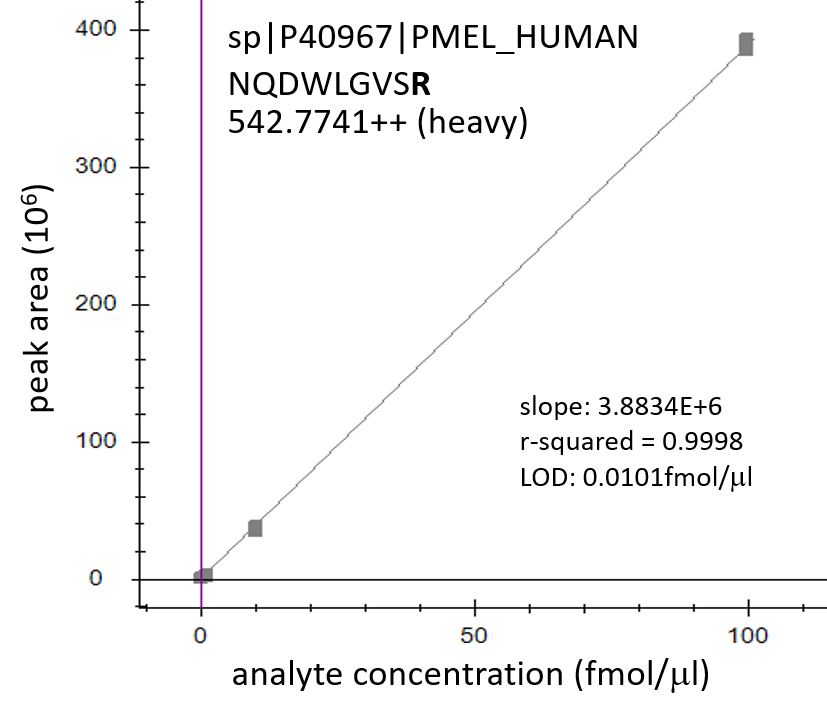


##### Fig. S5. Calibration curve of the PMEL peptide NQDWLGVSR

The calibration curve for the peptide NQDWLGVSR includes triplicate measurements of the AQUA peptide at 0.01 fmol, 0.01 fmol, 1 fmol, 10 fmol, and 100 fmol concentration and a blank reference. The calculated LOD of NQDWLGVSR (blank plus 2xSD) is 0.0101 fmol/μl.
